# From Crisis to Opportunity: Reinventing Medical Education After COVID-19

**DOI:** 10.5334/pme.1672

**Published:** 2025-06-27

**Authors:** Anita Samuel, Steven J. Durning

**Affiliations:** 1 Uniformed Services University of Health Sciences, US

## Abstract

The COVID-19 pandemic dramatically disrupted traditional bedside teaching in medical education, emphasizing the need for innovative approaches to clinical training. This disruption highlights a critical opportunity to reassess and enhance medical education practices for future resilience. This paper examines the changes in bedside teaching brought about by the COVID-19 pandemic and explores the use of novel tools and technologies to support this essential educational practice.

Key adaptations to bedside teaching involved virtual patient interviews, telemedicine clinics, and live-streamed surgeries. Hybrid models allowed for the co-location of some participants while integrating virtual supervision. Challenges included access disparities, technological limitations, and the inability to fully replicate hands-on training. Evidence supports the retention of traditional bedside teaching for high-contact encounters, hybrid models for limited-contact scenarios, and fully virtual teaching for non-contact educational needs.

The pandemic has demonstrated the adaptability and resilience of medical education. By strategically incorporating virtual and hybrid approaches, we can enhance the quality, accessibility, and effectiveness of bedside teaching. These approaches not only address the challenges posed by the pandemic but also offer opportunities to prepare future healthcare professionals for a dynamic and technologically advanced healthcare environment.

The COVID-19 pandemic forced medical education to undergo a rapid and unprecedented transformation towards online and hybrid learning modalities, challenging traditional bedside teaching models and necessitating innovative approaches to clinical training. This disruption, while jarring, presented an opportunity to “explore our capacity for adaptation amid rapid and constant change” [1, p. 2]. It has provided a space for a re-evaluation of traditional practices and the adoption of innovative approaches. The widespread adoption of telehealth, virtual simulations, and other digital tools not only addressed immediate challenges but also holds the potential to reshape the future of medical education, demanding a critical evaluation of which adaptations should be retained, refined, and discarded.

This period of profound change compels us to reflect on the current state of medical education, particularly in the context of bedside teaching. Bedside teaching is defined as “teaching when the patient is present” encompassing diverse healthcare environments [[Bibr B2][Bibr B3]]. Thus, bedside teaching includes hospital wards, outpatient clinics, and even the emergency room where the patient, teacher, and learner are present, with instruction occurring in the presence of the patient (i.e., the bedside). Physical distancing guidelines, restrictions on in-person gatherings, and the ubiquitous presence of personal protective equipment created barriers to the once-commonplace interactions between patients, learners, and clinical teachers [4,5]. While the pandemic impacted medical education across the continuum, bedside teaching, with its reliance on physical presence and direct observation, was particularly affected. In this environment, educators were forced to adapt, leveraging new tools and technologies to ensure the continuation of both patient care and learner education.

In this paper, we examine the specific changes observed in clinical education during the COVID-19 pandemic, focusing on how novel tools and technologies can be effectively employed to support bedside teaching. We explore how these adaptations can empower educators to overcome future obstacles, maintain learner engagement, and ensure the continuity of clinical education in the face of public health crises or other unforeseen disruptions. Finally, we offer evidence-based recommendations for optimizing medical education, outlining what practices should be initiated, continued, discontinued, and modified to enhance the quality, adaptability, and resilience of clinical training.

While the adoption of technology into medical education was necessitated and accelerated by the pandemic, it is important to acknowledge that many of the underlying technologies were not novel. Telemedicine platforms, virtual simulation tools, and digital communication channels were already available but remained largely underutilized in medical education [6,7]. Several factors may have contributed to this. Hands-on training and direct patient interaction are key features of medical education, and educators and students were reluctant to move away from familiar traditional teaching methods [8]. This was exacerbated by concerns about the effectiveness of virtual and hybrid modalities in replicating the in-person experience [9]. Prior to the pandemic, there was insufficient investment in technology resources in many medical schools leading to a lack of adequate infrastructure and support for technology integration [9].

## Theoretical Approach

**Figure 1:**
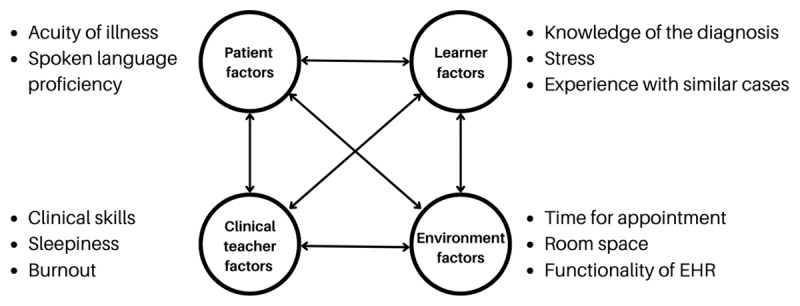
A situated cognition approach to bedside teaching.

We utilize situated cognition theory to frame our findings and recommendations for bedside teaching. This theory posits that learning is not solely an individual cognitive process but is deeply embedded within the specific context of the learning environment. “Situated cognition emphasizes the web of social and activity systems within which authentic practice takes shape” [10, p. 2]. In the case of bedside teaching, this environment comprises the dynamic interplay between the patient, clinical teacher, learner, and the physical and social affordances of the clinical setting (Figure 1). This framework highlights the critical role of ‘relationship-centered learning,’ where effective communication, collaboration, and mutual understanding between these key actors are essential for both optimal patient care and medical education [11]. By understanding how these elements interact and influence learning, we can better identify strategies to enhance bedside teaching, particularly in the face of disruptions and evolving healthcare landscapes. While some critics argue that situated cognition may overemphasize the role of context and underemphasize individual cognitive processes, its focus on dynamic interactions provides a valuable lens for analyzing the complexities of bedside teaching and informing practical recommendations for educators.

## Impact of COVID-19 on Bedside Teaching: Moving from Traditional Bedside Teaching to Fully Virtual and Hybrid Bedside Teaching

In the traditional bedside teaching model, the clinical teacher, patient, and learner are all physically present in the same location. Technology plays a supporting role in this environment, primarily through tools like electronic health records, clinical images, and patient monitoring devices, which facilitate communication and diagnosis. This traditional model emphasizes direct observation and hands-on practice, creating a learning experience that is inherently tied to a specific time and place. However, the restrictions imposed by the COVID-19 pandemic, such as social distancing mandates, disrupted this traditional model, necessitating a reimagining of bedside teaching.

During the pandemic, technology moved from being one component of the clinical encounter to an integral component. Utilizing technology in different ways, two variations of bedside teaching emerged: fully virtual bedside teaching and hybrid bedside teaching (see Table 1). These innovations in redesigning bedside teaching typically leveraged synchronous communication technologies that connected clinical teachers, patients, and learners across geographical distances. Some examples of synchronous technologies used are videoconferencing platforms such as Zoom and Google Meet, live-streaming platforms such as YouTube, and instant messaging services like WhatsApp.

In fully virtual bedside teaching, geographical and physical separation places the patient, clinical teacher, and learner in different locations. Technology was required to enable interactions. Therefore, patients, clinical teachers, and learners engaged with each other through synchronous technology-mediated interactions such as video calls, live streaming, or phone calls.

Fully virtual bedside teaching during the pandemic included telehealth consultations. Medical students conducted these consultations under the supervision of attending physicians (fully licensed doctors who have completed their residency training) or independently engaged with the patient to obtain the patient’s history and conducted virtual physicals [4,12,13,14,15,16,17]. In some instances, medical students participated independently in telemedicine clinics. Students were trained to conduct post-discharge follow-ups over the telephone, given patient encounter scripts, and had an attending on-call to answer questions in case of emergencies [4,18–19]. Patient encounter scripts allowed medical students to conduct patient follow-ups and participate in patient care [4,18–19]. Virtual rounds also required preparation before patient encounters. Preparations such as “practicing how to use the EHR (electronic health record) to identify relevant data for rounding, learning how to incorporate new data into oral presentations, and learning how to incorporate medical resources into oral presentations” were deemed very helpful by medical students [20, p. 5].

Sometimes, groups of students conducted patient interviews together virtually despite being geographically dispersed [21]. At other times, medical students were passive observers of telehealth consultations conducted by attendings [15,16,22]. Fully virtual bedside teaching was also conducted through online simulated clinical encounters. For example, learners reviewed simulated electronic health records, used simulated virtual patients, and conducted online virtual medical interviews [23]. Sometimes, learners assumed the roles of interviewer and standardized patients [24]. Fully virtual bedside teaching created opportunities for international collaborations. Countries collaborated on virtual clinical simulations through synchronous videoconferencing sessions [25,26].

All these fully virtual bedside teaching examples utilize videoconferencing platforms to create in-person interactions. However, international collaborations during the pandemic acknowledged that not all learners could access live-streaming technologies such as videoconferencing platforms, microphones, video cameras, broadband access, etc. Medical schools also experienced resource constraints. Therefore, elements of bedside encounters such as reading electronic health records were conducted via low-cost, low-resource simulations such as Simulation via Instant Messaging – Birmingham Advance (SIMBA) conducted entirely on WhatsApp [27].

## Hybrid Bedside Teaching

Hybrid bedside teaching combines traditional bedside teaching with fully virtual bedside teaching. Of the three human factors—clinical teacher, learner, and patient—two are co-located, and one is at a distance. Technology is required for interactions with the virtual participant.

Variations of hybrid bedside teaching were used during the pandemic. In variation 1, learners attended patient consultations virtually while the clinical teacher and patient were in the clinic (see Table 1) [28]. A similar approach was adopted for virtual rounds. Students participated in morning rounds and discussions on patients using mobile phones, virtual videoconferencing or telemedicine platforms, or mixed reality headsets worn by the clinical teacher [20,29,30,31,32,33]. Learners also attended surgeries and patient consultations via head-mounted cameras worn by the clinician. Learners could view the surgeries and consultations in real-time and converse with the surgeon [19]. Live streaming bedside consultations between the clinical teacher and patient also enabled participation from learners from around the globe [33].

**Table 1:** Fully Virtual and Hybrid Bedside Teaching.


FULLY VIRTUAL BEDSIDE TEACHING	HYBRID BEDSIDE TEACHING	

	VARIATION 1	VARIATION 2

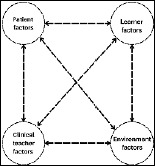 Patient, clinical teacher, and learner are in different locations.	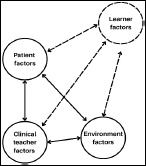 Patient and clinical teacher are in the same location while the learner is in another location.	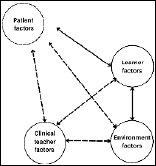 Patient and learner are in the same location while the clinical teacher is in another location.


The dotted lines indicate the geographic separation of all factors in the situated cognition model.

Variation 2 of hybrid bedside teaching placed the clinical teacher at a distance while the learner interacted with the patient in person (see Table 1). Invasive bedside procedures and surgeries adopted variation 2 hybrid bedside teaching strategies with residents (doctors who have graduated from medical school and are receiving specialized training and are supervised by attending physicians). Learners engaged with the patients during bedside procedures or consultations in the clinic and were supervised by attendings who were sometimes outside the patient room, participating using a mobile tablet-based camera system or videoconferencing systems [34,35].

Although virtual and hybrid models provide valuable learning opportunities, they cannot fully replace the hands-on experience, connection with the patient, and, at times their family (e.g., direct patient interaction) that characterize traditional bedside teaching.

## Interplay of Situated Cognition in the Three Teaching Modalities

We provide a case scenario to explicate how situated cognition is enacted across the three teaching modalities of traditional, fully virtual, and hybrid bedside teaching.

## Scenario

**Setting**: A teaching hospital’s cardiology ward.

**Participants**: A third-year medical student, a cardiology attending physician, and a patient who has recently been admitted with chest pain due to a myocardial infarction.

**Objective**: The main educational goal is for the student to learn how to perform a focused history and physical examination and to understand the clinical presentation and differential diagnosis of myocardial infarction.

**Preparation**: Before approaching the patient, the attending discusses the patient’s symptoms and findings with the student. They review the key elements of the history and the cardiovascular examination, emphasizing what findings might be relevant given the patient’s symptoms and findings.

**Bedside Interaction**: The attending introduces the medical student to the patient, ensuring to maintain professionalism and patient comfort. The student then conducts an abbreviated history and physical examination under the close supervision of the attending. After the history and physical examination, the attending discusses the key symptoms and findings with the student at the bedside. The attending and student might consult the EHR or other electronic monitors for further information.

Post-bedside debriefing: The attending asks the student to interpret the symptoms and findings and suggest a diagnosis and management plan. The attending physician provides feedback and corrects any mistakes in technique or interpretation. The attending elaborates on the significance of each symptom and finding and discusses potential management strategies. This interaction is also an opportunity to discuss how to communicate effectively with patients about their diagnosis and management plans.

Finally, the attending asks the student to reflect on what they learned during the session and how they can apply this knowledge in future clinical situations. The student is encouraged to ask questions and express any uncertainties they have about the case.

### Traditional Bedside Teaching

**Figure 2:**
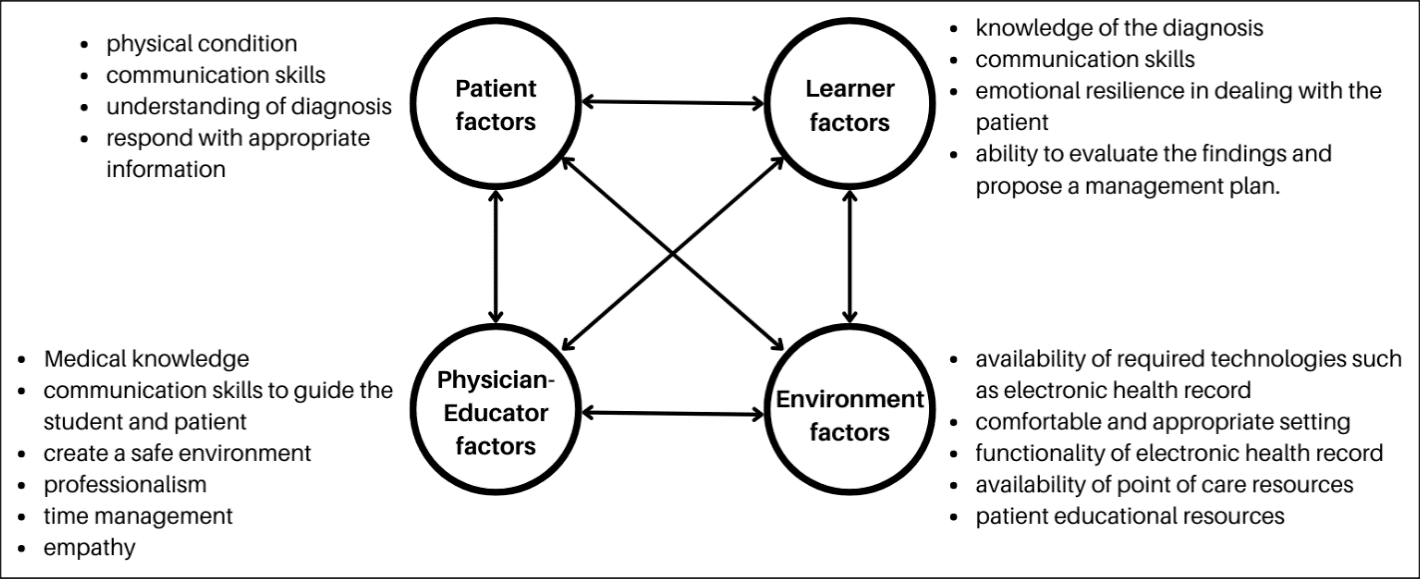
Situated cognition factors at play in a bedside teaching scenaro.

When the learner, patient, and educator are located in the same place, the factors that affect them are primarily related to the immediate clinical environment, direct interactions and communication between the learner, patient, and educator, including the patient’s condition and understanding, the learner’s skills and emotional resilience, and the educator’s guidance and ability to create a safe learning environment. See Figure 2 for a detailed overview of situated cognitive factors at play.

### Fully Virtual Bedside Teaching

**Figure 3:**
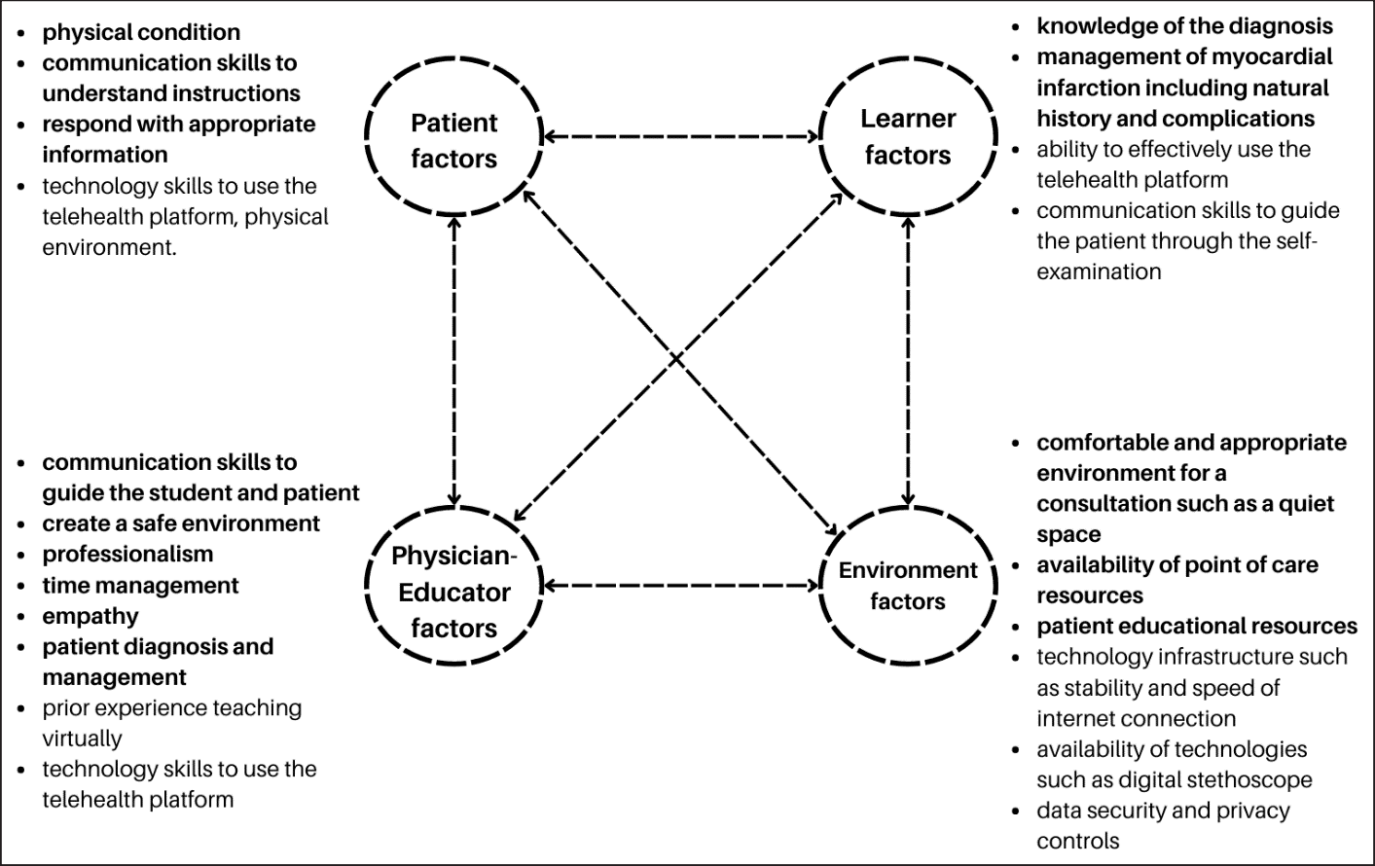
Points in bold are the same as in traditional bedside teaching.

Successful virtual encounters rely on robust technology infrastructure, including stable internet connectivity and appropriate devices. The patient’s comfort with technology and their physical environment, as well as the learner’s knowledge, communication skills, and emotional resilience, are crucial. The physician-educator must be proficient in virtual teaching, able to create a safe learning environment, and possess strong diagnostic and management skills. Access to resources, such as point-of-care information and patient educational materials, further enhances the virtual experience. Ultimately, optimizing virtual bedside teaching requires a thoughtful consideration of these interconnected factors to ensure effective learning and patient care (see Figure 3).

### Hybrid Bedside Teaching

A hybrid teaching approach requires technology skills in addition to being able to manage the physical and virtual environments. This model necessitates a balance between in-person experience and virtual guidance, requiring adaptability and effective communication from all participants (see Figure 3).

While the shift to virtual and hybrid bedside teaching modalities during the pandemic presented new technological and spatial dynamics, a situated cognition analysis reveals a common thread: the critical importance of communication. Whether in the traditional bedside setting or through a screen, effective communication between the physician, patient, and learner remains paramount for successful clinical encounters. Technology, while important, primarily serves to facilitate these interactions. Furthermore, the situated cognition lens highlights the interconnectedness of all participants and the environment, recognizing that various factors can influence the quality of care. Ultimately, fostering strong communication and recognizing the patient within a broader context of relationships remains crucial across all bedside teaching modalities.

## Lessons Learned

Bedside teaching is a valuable practice that allows learners to develop essential skills like communication, professionalism, clinical decision-making, and conducting histories and physical examinations. It also provides a unique opportunity for educators to mentor learners in real-time patient interactions. However, the literature suggests that this practice has been declining, with patient encounters increasingly taking place in conference rooms rather than at the bedside [36]. This shift is attributed to factors like increased patient volumes, time constraints, and reliance on medical testing and imaging [36,37,38,39]. The technological innovations triggered by the COVID-19 pandemic offer opportunities for us to increase direct patient interactions.

Bedside teaching encounters can be categorized based on the physical contact required with the patient. They can be very minimal to no contact, limited contact, or high contact. Each type of encounter is designed to meet specific clinical needs while considering the level of physical contact necessary for effective patient care. While the concept of varying levels of physical contact in clinical encounters is inherent in medical practice, a formal categorization based on contact level has not been explicitly codified in the medical education literature. We have created a categorization and base this categorization on a contemporary theory. Each type of encounter is designed to meet specific clinical needs while considering the level of physical contact necessary for effective patient care. Classifying bedside teaching encounters based on contact level grounded in established theory allows educators to tailor their teaching to specific clinical needs and learning objectives, optimize resource allocation, and enhance safety. Table 2 provides examples of these encounters.

**Table 2:** Types of Clinical Encounters.


TYPE OF ENCOUNTER	HIGH CONTACT ENCOUNTERS	LIMITED CONTACT ENCOUNTERS	VERY MINIMAL TO NO CONTACT ENCOUNTERS

**Purpose of Encounter**	To perform procedures or interventions that require extensive physical interaction and often involve more invasive techniques.	To perform assessments or interventions that require minimal physical interaction with the patient.	To gather information, provide education, and discuss care plans without the need for physical examination or procedures.

**Examples of Encounter**	Surgical procedures (e.g., incisions, suturing) Invasive diagnostic tests (e.g., lumbar puncture, biopsies) Advanced cardiovascular assessments (e.g., cardiac catheterization, echocardiograms, stress tests) Emergency interventions (e.g., cardiopulmonary resuscitation, intubation) Rehabilitation exercises requiring physical assistance	Visual inspection (e.g., dermatologic inspection of rashes and other selected lesions) Measuring vital signs Basic neurological assessments – conducted with appropriate equipment (e.g., checking mental status, pupil response, grip strength)	Patient history taking Patient education and counseling Discussing selected diagnostic results and management options Limited psychological and emotional support Limited advance care planning and family meetings

**Suggested Teaching Modality**	**Traditional Bedside Teaching** 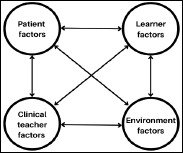	**Hybrid Bedside Teaching** 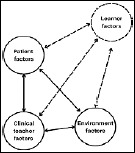	**Fully Virtual Bedside Teaching** 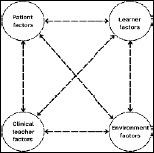


Different bedside teaching encounters are better suited to certain modalities. High-contact encounters need in-person interaction, and traditional bedside teaching is the best fit. Limited contact clinical encounters, such as visual inspections and measuring vital signs, can be conducted in hybrid bedside teaching encounters. Certain clinical encounters, such as history taking and patient education, can be conducted effectively from a distance. These very minimal to no contact encounters can be conducted completely virtually. By appropriately leveraging the technologies available, we can create more robust bedside teaching programs [40]. Table 3 presents the technology modalities that could be used for different clinical encounters.

**Table 3:** Balancing in-person and virtual bedside teaching scenarios.


	HIGH CONTACT ENCOUNTERS	LIMITED CONTACT ENCOUNTERS	VERY MINIMAL TO NO CONTACT ENCOUNTERS

**Keep Doing**	Traditional bedside teaching	Traditional bedside teaching	

**Start Doing**		Hybrid bedside teaching (e.g., virtual observation of in-person encounters, remote supervision of procedures)	Fully virtual bedside teaching (e.g., telemedicine clinics, virtual patient interviews)

**Stop Doing**			Traditional bedside teaching for non-contact encounters

**Rationale**	Essential for hands-on skills and nuanced interactions	Offers a balance of in-person experience and virtual flexibility	Optimizes resources and expands access to diverse patient populations


Very minimal to no contact patient encounters such as history taking and, at times, patient education and counseling, advance care planning, and/or family meetings could be conducted completely virtually. This approach has some advantages. Learners can engage with more patients while they learn key teleconsultation communication skills. These virtual encounters reduce the need for patients to travel, while educators can directly observe learner skills.

Medical encounters involving limited contact, like visual inspections (e.g., rashes), measuring vital signs, or, at times, administering oral medication, can be effectively conducted in fully virtual or hybrid settings. Fully virtual encounters require reliable technology, such as high-quality cameras, precise measurement tools, and well-prepared patients. Routine check-ins, where patients are equipped with necessary devices and trained in telemedicine, are ideal for fully virtual consultations. However, initial encounters may be more suitable in a hybrid format, where the patient is physically present with either the learner or the clinical teacher.

Bedside teaching involving high levels of contact necessitates in-person interaction. Essential skills like physical examinations, surgical procedures, and diagnostic tests demand hands-on practice for effective learning. While videos and simulations offered temporary alternatives during the pandemic, they cannot fully replace the invaluable experience of direct, in-person training. The experience during the pandemic reinforced the importance of maintaining traditional bedside teaching for high-contact encounters, where direct patient interaction and hands-on practice are essential for developing critical clinical skills.

The patient encounter in bedside teaching is complemented by lectures, small group discussions, journal clubs, case discussions, etc., to reinforce clinical learning [36]. During the pandemic, a plethora of technologies were used to transition these educational interactions to a virtual format. Videoconferencing technologies enabled educators to deliver lectures and conduct small group discussions virtually. Case reviews, grand rounds, and resident lectures also adopted these platforms [22,36,37,38,40]. Demonstration of clinical skills and theoretical concepts were shared via videos, podcasts, social media, open-access websites, and online learning modules [23,41,42,43,44,45,46]. Skills such as using electronic health records can be taught virtually via simulations so learners can practice in a safe environment before entering the clinical environment [47]. The COVID-19 pandemic has shown that knowledge transfer teaching can be done virtually and asynchronously. Continuing this practice as we move forward will allow learners more time to practice hands-on skills in the clinic.

## Looking Ahead

The largely successful transformations discussed above should not be just a response to a temporary crisis; we should strategically incorporate such innovations and shift towards preparing future medical professionals for a dynamic and unpredictable healthcare environment. Certain traditional approaches to bedside teaching must be maintained (‘keep doing’) while we can move away from others (‘stop doing’) and consider some new approaches (‘start doing’). Appropriate modifications can help us reduce the challenges to traditional bedside teaching.

### Keep Doing

While fully virtual and hybrid bedside teaching were adopted globally to ensure ongoing education, these technologies could not fully recreate a clinical encounter. Learners could not assist with hands-on procedures or practice motor skills [25,28]. While virtual reality (VR) technologies attempt to recreate hands-on procedures, they still fall short of in-person interactions. We recommend that we keep traditional bedside teaching for high-contact patient encounters. Traditional bedside teaching must be preserved for high-contact encounters, where the development of hands-on skills and the ability to navigate complex patient interactions are paramount.

### Start Doing

During the pandemic, medical students were an invaluable asset. They supported patient follow-up, education, and triage, alleviating the burden on overworked healthcare professionals [18,48–49]. As healthcare faces an increasing shortage of healthcare professionals and learners have insufficient patient contact in clinical rotations, we should explore and enhance the potential of learners as “clinicians-in-training” who can “interview patients, call consults, respond to pages, communicate with families, write notes, assist with procedures, and help with care coordination and discharge planning” [50, p. 1].

Telemedicine clinics and live-streamed surgery sessions allowed learners to participate in specialty clinics and exposed them to a broader range of patient populations and pathophysiology [38,49,51]. These sessions permitted learners to view examinations and surgeries that would otherwise not be possible [22]. These opportunities could be offered to complement learners’ clinical rotations. By continuing to leverage the technological innovations forced by the pandemic, we can create more accessible and inclusive learning environments. These innovations exposed learners to more clinical experiences and global experts and opened new possibilities.

All the educational innovations during the pandemic were predicated on technological affordances. These innovations can be continued with robust technology infrastructure and training for clinical teachers, patients, and learners. Training and institutional infrastructure need to be formalized in the medical education curriculum.

### Stop Doing

In addition to revealing new possibilities, the pandemic also highlighted the limitations of some traditional pedagogical strategies. Recorded lectures, videotaped demonstrations, podcasts, online learning modules, and webinars can, at times (e.g. low engagement lectures), replace in-person didactic lectures and have proven to be more effective than traditional lectures. They also allow for the global sharing and reuse of content, global access to healthcare expertise, and reduced travel and scheduling complexities [22]. Lectures that are a one-way delivery of content can be recorded and shared with learners. Precious class time can be reserved for active learning through case studies, high-engagement lectures, group discussions, and labs.

### Acknowledging Limitations

Bedside encounters cannot always be cleanly distinguished as involving very limited to no contact, minimal contact, or high contact encounters. Sometimes, encounters can involve a mix of these within a single visit. Thoughtful preparation and triage can help in deciding the best form of bedside encounter to adopt. Prior to any encounter, a careful review of the patient’s history and presenting concerns allows for a determination of the necessary level of contact. If a physical examination or high-contact interaction is deemed essential, appropriate in-person appointments can be scheduled, ensuring that the necessary resources and precautions are in place. If the encounter can be managed with limited or no contact, telehealth tools or modified in-person approaches, such as guided self-examination, can be utilized, optimizing resource allocation and minimizing unnecessary exposure. This pre-encounter planning allows for efficient and safe patient care, tailoring the approach to the patient’s specific needs.

In summary, technology was the innovation during the pandemic. Using technology, new and original solutions to problems were explored, efficiency was improved, and medical education was transformed. Table 3 provides a snapshot of what we propose for medical education moving forward. High contact and certain limited contact clinical encounters must use traditional bedside teaching. Technology is not yet at the point of providing a comparable alternative. However, we should start using hybrid and fully virtual bedside teaching for non-contact and certain limited contact encounters. To address the human resource challenges in healthcare, we need to stop using valuable traditional bedside teaching time on non-contact encounters that can be effectively conducted online.

## Conclusion

The COVID-19 pandemic has undeniably disrupted traditional bedside teaching practices. However, it has also catalyzed a technological revolution in medical education, offering innovative solutions to enhance learner-patient interactions. Pre-COVID, technology was part of the environment and connective tissue for situated cognition, facilitating communication (EHR, email, etc.). Post-COVID, technology itself is the main innovation. We can create a fit-for-purpose, comprehensive, and effective bedside teaching program by strategically integrating virtual, hybrid, and in-person modalities. This adaptive approach accommodates the diverse nature of medical encounters and optimizes the learning experience for medical students and residents. This approach acknowledges that while virtual and hybrid approaches have enriched medical education, traditional in-person bedside teaching remains a cornerstone of clinical training, providing irreplaceable experiences that shape the development of skilled and compassionate healthcare professionals. As we move forward, embracing these advancements will be crucial in shaping the future of medical education and fostering a new generation of skilled and compassionate healthcare professionals.

## Disclaimer

The contents of this publication are the sole responsibility of the authors and do not necessarily reflect the views, assertions, opinions, or policies of the Uniformed Services University of the Health Sciences (USUHS) or the Departments of Defense.
